# Longitudinal follow-up study of the association with gout and Alzheimer’s disease and Parkinson’s disease in Korea

**DOI:** 10.1038/s41598-023-30379-4

**Published:** 2023-03-06

**Authors:** Eun Jae Lee, So Young Kim, Hyo Geun Choi, Yoo Hwan Kim, Mi Jung Kwon, Joo-Hee Kim, Heui Seung Lee, Jae Keun Oh, In Bok Chang, Joon Ho Song, Ji Hee Kim

**Affiliations:** 1 College of Nursing, Ewha Womans University, Seoul, 03760 Korea; 2 CHA Bundang Medical Center, Department of Otorhinolaryngology-Head and Neck Surgery, CHA University, Seongnam, 13496 Korea; 3 MD Analytics, Seoul, 06349 Korea; 4 Department of Otorhinolaryngology-Head and Neck Surgery, Suseoseoul ENT Clinic, Seoul, 06349 Korea; 5 Department of Neurology, Hallym University College of Medicine, Anyang, 14068 Korea; 6 Department of Pathology, Hallym University College of Medicine, Anyang, 14068 Korea; 7 Division of Pulmonary, Allergy, and Critical Care Medicine, Department of Medicine, Hallym University College of Medicine, Anyang, 14068 Korea; 8 Department of Neurosurgery, Hallym University College of Medicine, Anyang, 14068 Korea

**Keywords:** Diseases, Medical research, Neurology, Pathogenesis, Rheumatology

## Abstract

To date, no clear conclusion on the relationships of gout with the occurrence of typical neurodegenerative diseases, Alzheimer’s disease (AD) and Parkinson’s disease (PD), has been reached. This study aimed to determine whether the patients with gout are at a lower or higher probability of developing AD or PD than those without gout. Longitudinal follow-up data of a representative sample of Korean adults were assessed. 18,079 individuals diagnosed with gout between 2003 and 2015 were enrolled in the gout group. The comparison group comprised 72,316 demographics-matched individuals not diagnosed with gout. Longitudinal associations of gout with AD or PD were estimated using Cox proportional hazard regression adjusting for potential confounders. The adjusted hazard ratios (HRs) of AD and PD in the gout group were 1.01 and 1.16 times higher than controls, but these differences were not statistically significant (95% confidence interval [CI] = 0.92–1.12 and 95% CI = 0.97–1.38, respectively). Although there was no significant association in the entire sample, AD and PD probabilities in patients with gout were significantly higher in participants < 60 years, and PD probabilities in patients with gout were significantly higher in overweight participants. Our findings identify significant correlations of gout with AD and PD in participants < 60 years and gout with PD in those with overweight, indicating that gout may play a role in the development of neurodegenerative diseases in younger or overweight populations. Further investigations should be performed to corroborate these findings.

## Introduction

Gout is a common systemic inflammatory disease characterized by severe joint pain. It results from the deposition of monosodium urate crystals in the synovial fluid of joints and in other tissues caused by a disorder of purine metabolism that leads to hyperuricemia. Gout presents either as recurrent acutely painful arthritis in a few joints or as chronic inflammatory polyarthritis affecting small and large joints of the extremities, which significantly impairs patients’ health-related quality of life. Hyperuricemia is the result of an increased production of uric acid, hypoexcretion of uric acid by the kidneys, or both^1^.

Alzheimer’s disease (AD), the most frequent form of dementia, is an irreversible, progressive neurodegenerative disorder of the central nervous system that is characterized by impairments of memory and learning. Its pathogenesis has been attributed to extracellular aggregations of beta-amyloid plaques and intracellular neurofibrillary tangles consisting of hyperphosphorylated tau protein^2^. Although numerous risk factors for AD have been suggested, it remains unknown whether any of these factors lead to amyloid deposition and tauopathy in humans. Parkinson’s disease (PD) is the second most common neurodegenerative disorder; it produces not only cardinal motor symptoms but also a wide range of nonmotor symptoms. Although significant progress has been made in elucidating the pathogenesis of PD, the cause of the progressive degeneration of dopaminergic neurons in the substantia nigra and the pathologic characteristics of PD remain unclear.

Because uric acid is a potent natural antioxidant and oxidative stress is one of the major pathogenic mechanisms of neurodegenerative diseases, close associations between gout due to hyperuricemia and neurodegenerative diseases, such as AD and PD, seem highly plausible. However, regarding whether gout, with its associated high serum levels of uric acid, protects against neurodegenerative disorders remains debatable. Some studies have reported that serum uric acid is not related to a lower risk of AD^3^; in contrast, a study of the United Kingdom Health Improvement Network reported that the multivariate-adjusted hazard ratio (HR) for AD among gout patients was 0.71 (95% confidence interval [CI] = 0.62–0.80), implicating a meaningful protective effect^4^. Similar to the findings of AD, studies on the relationship between gout and PD have also reached mixed conclusions^5,6^. One UK study using a large population-based database concluded that individuals with a previous history of gout had a lower risk of developing PD (odds ratio [OR] = 0.69, 95% CI = 0.48–0.99)^5^. In contrast, a report derived from US Medicare claims data showed that gout was associated with a 14% increased risk of incident PD^6^. Overall, epidemiological studies have reported conflicting results. Moreover, recent data have called into question the neuroprotective role of uric acid; therefore, the association between gout and neurodegenerative diseases remains equivocal^7,8^.

We thus aimed to determine the associations of gout with incident AD and PD in a Korean population through a longitudinal follow-up study, adjusting for various potential confounders (including metabolic syndrome-related risk factors).

## Results

Although there was a minor difference in the distribution of obesity between the gout group and the comparison group (standardized difference = 0.27), the absolute value of the standardized difference for most variables was less than 0.2, indicating that the intergroup differences in most baseline characteristics were well balanced after matching (Table 1).

**Table 1:** General characteristics of participants.

Characteristics	Total participants		
	Gout (n, %)	Control (n, %)	Standardized difference
Age (years old)			0.00
40–44	579 (3.2)	2316 (3.2)	
45–49	2049 (11.3)	8196 (11.3)	
50–54	3460 (19.1)	13,840 (19.1)	
55–59	3357 (18.6)	13,428 (18.6)	
60–64	2826 (15.6)	11,304 (15.6)	
65–69	2476 (13.7)	9904 (13.7)	
70–74	1838 (10.2)	7352 (10.2)	
75–79	1062 (5.9)	4248 (5.9)	
80–84	357 (2.0)	1428 (2.0)	
85+	75 (0.4)	300 (0.4)	
Sex			0.00
Male	14,490 (80.1)	57,960 (80.1)	
Female	3589 (19.9)	14,356 (19.9)	
Income			0.00
1 (lowest)	2514 (13.9)	10,056 (13.9)	
2	2235 (12.4)	8940 (12.4)	
3	2753 (15.2)	11,012 (15.2)	
4	3805 (21.0)	15,220 (21.0)	
5 (highest)	6772 (37.5)	27,088 (37.5)	
Residential area			0.00
Urban	7677 (42.5)	30,708 (42.5)	
Rural	10,402 (57.5)	41,608 (57.5)	
Obesity^†^			0.27
Underweight	240 (1.3)	1810 (2.5)	
Normal	4548 (25.2)	25,158 (34.8)	
Overweight	4978 (27.5)	20,141 (27.9)	
Obese I	7573 (41.9)	23,459 (32.4)	
Obese II	740 (4.1)	1748 (2.4)	
Smoking status			0.08
Nonsmoker	10,315 (57.1)	40,314 (55.7)	
Past smoker	3567 (19.7)	12,869 (17.8)	
Current smoker	4197 (23.2)	19,133 (26.5)	
Alcohol consumption			0.09
< 1 time a week	9408 (52.0)	41,025 (56.7)	
≥ 1 time a week	8671 (48.0)	31,291 (43.3)	
Systolic blood pressure			0.13
< 120 mmHg	4226 (23.4)	19,927 (27.6)	
120–139 mmHg	8798 (48.7)	35,949 (49.7)	
≥ 140 mmHg	5055 (28.0)	16,440 (22.7)	
Diastolic blood pressure			0.13
< 80 mmHg	6769 (37.4)	30,597 (42.3)	
80–89 mmHg	6849 (37.9)	27,248 (37.7)	
≥ 90 mmHg	4461 (24.7)	14,471 (20.0)	
Fasting blood glucose			0.05
< 100 mg/dL	10,647 (58.9)	43,630 (60.3)	
100–125 mg/dL	5740 (31.7)	21,259 (29.4)	
≥ 126 mg/dL	1692 (9.4)	7427 (10.3)	
Total cholesterol			0.09
< 200 mg/dL	9388 (51.9)	40,304 (55.7)	
200–239 mg/dL	5979 (33.1)	23,181 (32.1)	
≥ 240 mg/dL	2712 (15.0)	8831 (12.2)	
Charlson comorbidity index			0.12
0	11,345 (62.8)	49,835 (68.9)	
1	2796 (15.5)	9979 (13.8)	
≥ 2	3938 (21.8)	12,502 (17.3)	
Alzheimer’s disease	519 (2.9)	1989 (2.8)	0.01
Parkinson’s disease	166 (0.9)	563 (0.8)	0.02

^†^Obesity (BMI, body mass index, kg/m^2^) was categorized as < 18.5 (underweight), ≥ 18.5 to < 23 (normal), ≥ 23 to < 25 (overweight), ≥ 25 to < 30 (obese I), and ≥ 30 (obese II).

**Figure 1:**
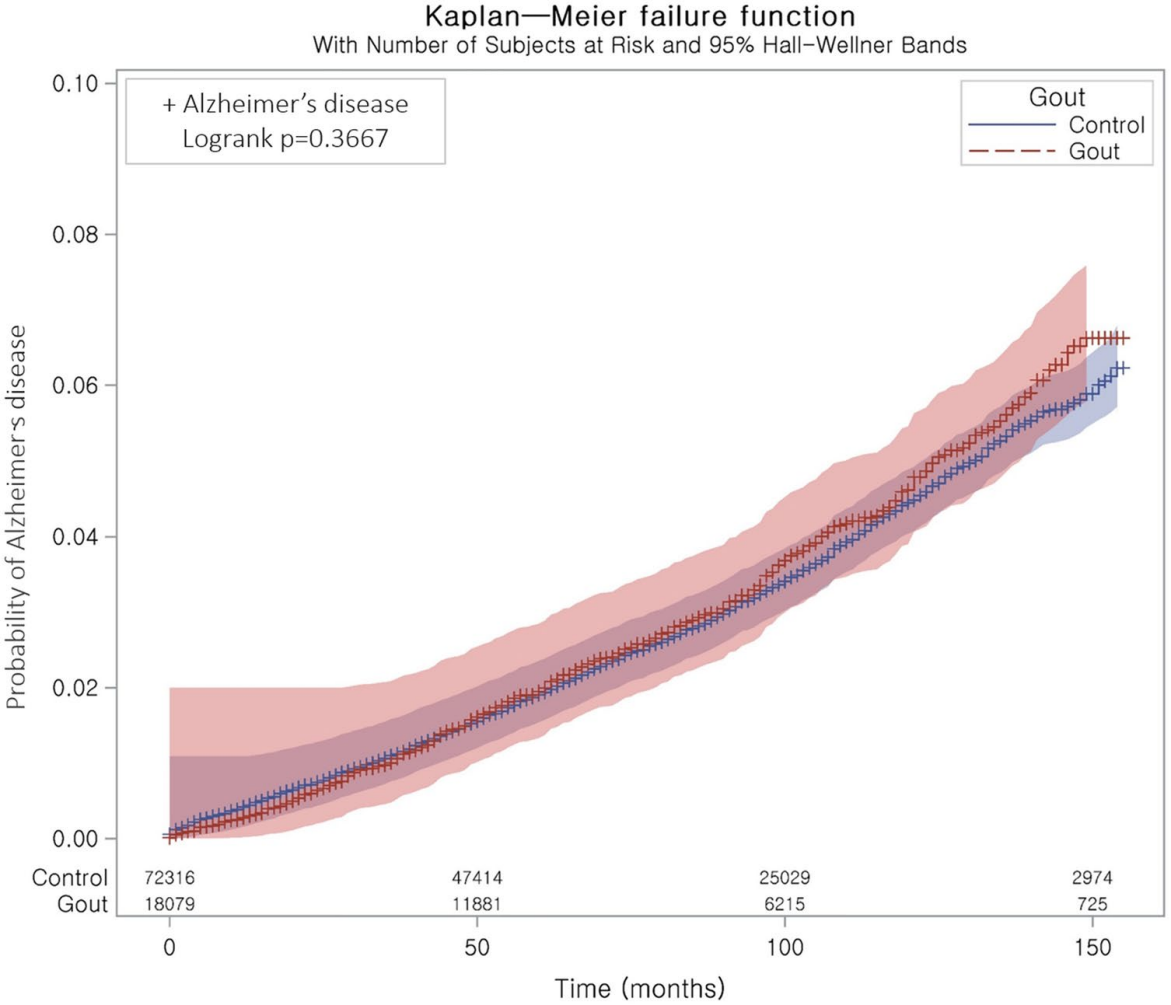
Kaplan‒Meier probability of the incidence of Alzheimer’s disease in gout and the control participants within 13 years of the index date.

The incidence rate of AD was 4.91 per 1000 person-years in the gout group and 4.71 per 1000 person-years in the comparison group. In patients with gout, the crude HR (matched on age, sex, income, and residential area) of AD was 1.05 (95% CI = 0.95–1.15, P = 0.360). After adjustment for obesity, smoking status, alcohol consumption, blood pressure, fasting blood glucose, total cholesterol, CCI score, and PD, the HR of AD during the follow-up period was 1.01 for patients in the gout group (95% CI = 0.92–1.12, P = 0.825, Table 2). The Kaplan–Meier survival curves and log-rank test of the gout and comparison groups indicated that the probability of AD was not significantly higher in the gout group than in the comparison group during the follow-up period (P = 0.367 in log-rank test, Fig. 1).

**Table 2:** Crude and adjusted hazard ratios of gout for Alzheimer’s disease with subgroup analyses stratified based on covariates.

	Incidence rate per 1000 person-years	Incidence rate difference per 1000 person-years (95% confidence interval)	Hazard ratio for Alzheimer’s disease (95% confidence interval)			
			Crude^†^	P-value	Adjusted^†‡^	P-value
Total participants (n = 90,395)						
Gout	4.91	0.21 (− 0.26 to 0.67)	1.05 (0.95–1.15)	0.360	1.01 (0.92–1.12)	0.825
Control	4.71					
Age < 60 (n = 47,225)						
Gout	0.90	0.36 (0.14 to 0.57)	1.65 (1.22–2.25)	0.001*	1.46 (1.07–2.00)	0.018*
Control	0.55		1		1	
Age ≥ 60 (n = 43,170)						
Gout	11.12	− 0.06 (− 1.19 to 1.08)	1.00 (0.90–1.11)	0.999	0.97 (0.87–1.08)	0.559
Control	11.17		1		1	
Men (n = 72,450)						
Gout	4.17	0.01 (− 0.48 to 0.49)	0.99 (0.88–1.11)	0.892	0.97 (0.86–1.09)	0.642
Control	4.16		1		1	
Women (n = 17,945)						
Gout	8.17	1.13 (− 0.20 to 2.46)	1.19 (1.00–1.42)	0.052	1.10 (0.92–1.31)	0.322
Control	7.04		1		1	
Underweight (n = 2050)						
Gout	7.49	− 2.59 (− 8.53 to 3.35)	0.73 (0.37–1.45)	0.371	0.61 (0.30–1.24)	0.174
Control	10.08		1		1	
Normal weight (n = 29,706)						
Gout	5.83	0.43 (− 0.55 to 1.42)	1.08 (0.91–1.29)	0.375	0.99 (0.83–1.19)	0.946
Control	5.40		1		1	
Overweight (n = 25,119)						
Gout	4.48	0.43 (− 0.39 to 1.26)	1.11 (0.91–1.34)	0.307	0.95 (0.78–1.15)	0.601
Control	4.05		1		1	
Obese (n = 33,520)						
Gout	4.63	0.43 (− 0.24 to 1.09)	1.10 (0.95–1.28)	0.204	1.10 (0.94–1.28)	0.236
Control	4.20		1		1	
SBP < 140 mmHg and DBP < 90 mmHg (n = 64,040)						
Gout	4.41	0.36 (− 0.18 to 0.90)	1.09 (0.96–1.24)	0.187	1.03 (0.90–1.17)	0.701
Control	4.05		1		1	
SBP ≥ 140 mmHg or DBP ≥ 90 mmHg (n = 26,355)						
Gout	5.75	− 0.42 (− 1.30 to 0.45)	0.93 (0.81–1.08)	0.351	1.00 (0.86–1.16)	0.979
Control	6.17		1		1	
Fasting blood glucose < 100 mg/dL (n = 54,277)						
Gout	4.38	0.20 (− 0.36 to 0.75)	1.05 (0.92–1.19)	0.483	1.01 (0.89–1.16)	0.840
Control	4.19		1		1	
Fasting blood glucose ≥ 100 mg/dL (n = 36,118)						
Gout	5.76	0.17 (− 0.65 to 0.99)	1.03 (0.89–1.19)	0.654	1.01 (0.87–1.17)	0.904
Control	5.59		1		1	
Total cholesterol < 200 mg/dL (n = 49,692)						
Gout	5.01	0.11 (− 0.56 to 0.77)	1.02 (0.90–1.17)	0.729	0.94 (0.83–1.08)	0.412
Control	4.91		1		1	
Total cholesterol ≥ 200 mg/dL (n = 40,703)						
Gout	4.82	0.35 (− 0.29 to 1.00)	1.08 (0.94–1.24)	0.286	1.10 (0.95–1.27)	0.186
Control	4.46		1		1	

*SBP* systolic blood pressure, *DBP* diastolic blood pressure. *Stratified or unstratified cox proportional hazard regression model, Significance at P < 0.05. ^†^Matched model based one age, sex, income, and residential area. ^‡^Adjusted for obesity, smoking status, alcohol consumption, SBP, DBP, fasting blood glucose, total cholesterol, Charlson comorbidity index scores, and Parkinson’s disease.

**Figure 2:**
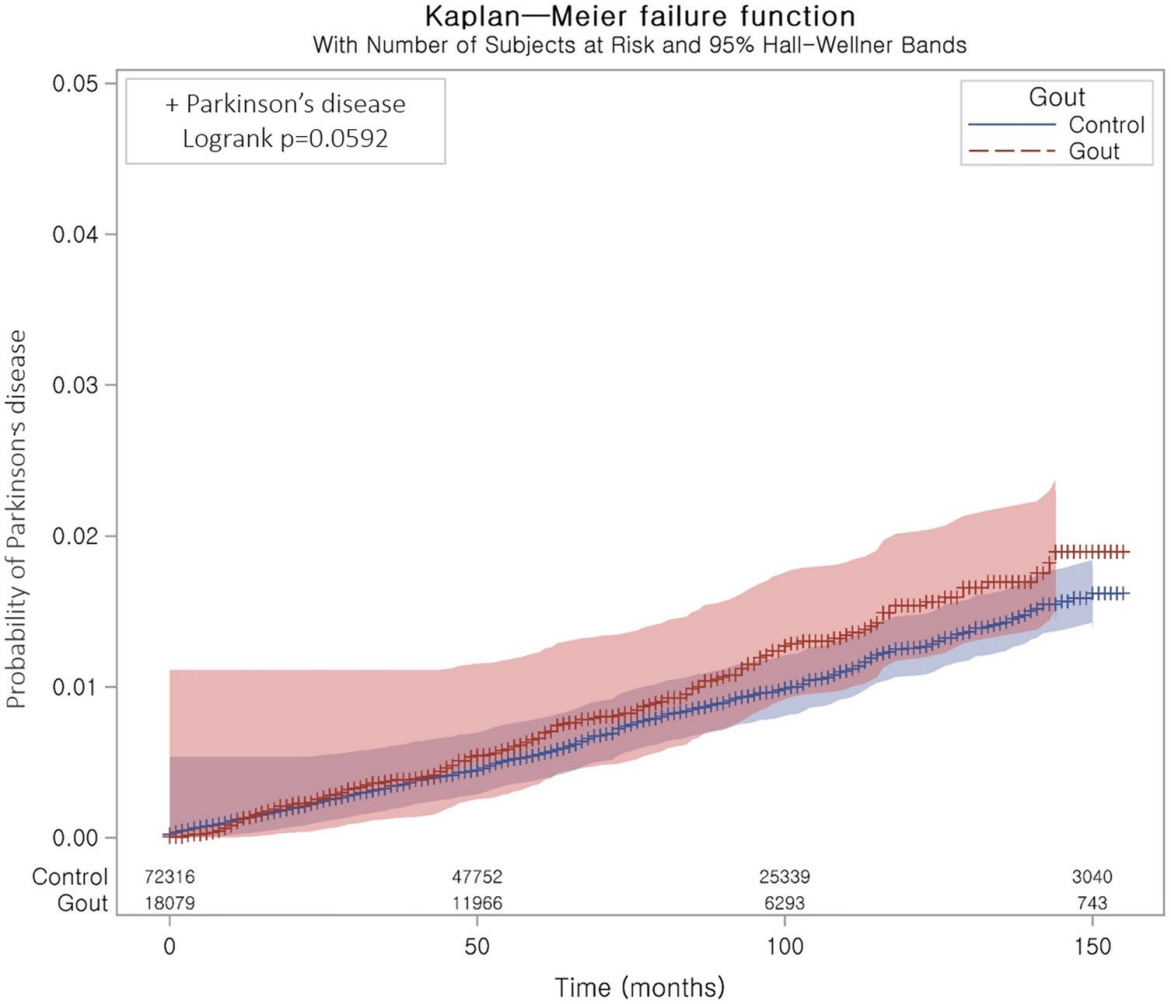
Kaplan‒Meier probability of the incidence of Parkinson’s disease in gout and the control participants within 13 years of the index date.

The incidence rate of PD (per 1000 person-years) was 1.56 in the gout group and 1.32 in the comparison group. The longitudinal association between gout and PD had a crude HR of 1.18 (95% CI = 0.99–1.41, P = 0.057) and an adjusted HR of 1.16 (95% CI = 0.97–1.38, P = 0.100, Table 3); these values were not statistically significant. In the analysis of the cumulative incidence of PD according to the presence of gout, no significant between-group differences were found (P = 0.059 in log-rank test, Fig. 2).

**Table 3:** Crude and adjusted hazard ratios of gout for Parkinson’s disease with subgroup analyses stratified based on covariates.

Independent variables and subgroup	Incidence rate per 1000 person-years	Incidence rate difference per 1000 person-years (95% confidence interval)	Hazard ratio for Parkinson’s disease (95% confidence interval)			
			Crude^†^	P-value	Adjusted^†‡^	P-value
Total participants (n = 90,395)						
Gout	1.56	0.24 (− 0.01 to 0.49)	1.18 (0.99–1.41)	0.057	1.16 (0.97–1.38)	0.100
Control	1.32		1		1	
Age < 60 (n = 47,225)						
Gout	0.56	0.22 (0.05 to 0.39)	1.63 (1.11–2.40)	0.013*	1.55 (1.04–2.31)	0.032*
Control	0.35		1		1	
Age ≥ 60 (n = 43,170)						
Gout	3.08	0.27 (− 0.30 to 0.84)	1.10 (0.91–1.33)	0.340	1.07 (0.88–1.31)	0.492
Control	2.81		1		1	
Men (n = 72,450)						
Gout	1.47	0.18 (− 0.10 to 0.45)	1.13 (0.93–1.38)	0.209	1.14 (0.93–1.39)	0.207
Control	1.30		1		1	
Women (n = 17,945)						
Gout	1.95	0.52 (− 0.09 to 1.12)	1.37 (0.95–1.97)	0.087	1.28 (0.89–1.86)	0.188
Control	1.44		1		1	
Underweight (n = 2050)						
Gout	4.14	1.73 (− 1.32 to 4.78)	1.70 (0.65–4.47)	0.282	1.37 (0.49–3.88)	0.551
Control	2.41		1		1	
Normal weight (n = 29,706)						
Gout	1.59	0.18 (− 0.32 to 0.68)	1.12 (0.80–1.57)	0.491	1.05 (0.75–1.48)	0.758
Control	1.41		1		1	
Overweight (n = 25,119)						
Gout	1.78	0.64 (0.18 to 1.10)	1.56 (1.13–2.14)	0.007*	1.41 (1.02–1.95)	0.037*
Control	1.14		1		1	
Obese (n = 33,520)						
Gout	1.35	0.05 (− 0.32 to 0.42)	1.04 (0.79–1.37)	0.786	0.99 (0.75–1.31)	0.966
Control	1.31		1		1	
SBP < 140 mmHg and DBP < 90 mmHg (n = 64,040)						
Gout	1.42	0.23 (− 0.06 to 0.53)	1.20 (0.95–1.50)	0.121	1.15 (0.91–1.45)	0.237
Control	1.19		1		1	
SBP ≥ 140 mmHg or DBP ≥ 90 mmHg (n = 26,355)						
Gout	1.79	0.17 (− 0.29 to 0.63)	1.10 (0.85–1.44)	0.464	1.16 (0.88–1.51)	0.293
Control	1.62		1		1	
Fasting blood glucose < 100 mg/dL (n = 54,277)						
Gout	1.50	0.23 (− 0.08 to 0.54)	1.18 (0.94–1.48)	0.143	1.12 (0.89–1.41)	0.331
Control	1.27		1		1	
Fasting blood glucose ≥ 100 mg/dL (n = 36,118)						
Gout	5.76	4.11 (3.62 to 4.59)	1.17 (0.89–1.54)	0.248	1.20 (0.91–1.57)	0.201
Control	1.65		1		1	
Total cholesterol < 200 mg/dL (n = 49,692)						
Gout	1.72	0.28 (− 0.08 to 0.65)	1.20 (0.95–1.51)	0.126	1.14 (0.90–1.44)	0.274
Control	1.43		1		1	
Total cholesterol ≥ 200 mg/dL (n = 40,703)						
Gout	1.41	0.22 (− 0.12 to 0.55)	1.18 (0.91–1.53)	0.207	1.15 (0.88–1.50)	0.302
Control	1.19		1		1	

*SBP* systolic blood pressure, *DBP* diastolic blood pressure. *Stratified or unstratified cox proportional hazard regression model, Significance at P < 0.05. ^†^Matched model based one age, sex, income, and residential area. ^‡^Adjusted for obesity, smoking status, alcohol consumption, SBP, DBP, fasting blood glucose, total cholesterol, Charlson comorbidity index scores, and Alzheimer’s disease.

To determine if covariates contributed to the nonsignificant relationships of gout with AD and PD, we further conducted subgroup analyses stratified by multiple covariates. Gout remained a nonsignificant factor for AD in all subgroups, except for the age subgroup of individuals < 60 years old (adjusted HR = 1.46, 95% CI = 1.07–2.00, P = 0.018, Table 2). Similarly, gout was not associated with the probability of PD in most covariates-stratified subgroups. A positive correlation of gout with PD remained only in patients < 60 years old (adjusted HR = 1.55, 95% CI = 1.04–2.31, P = 0.032) and in those who were overweight (adjusted HR = 1.41, 95% CI = 1.02–1.95, P = 0.037, Table 3).

## Discussion

In this large population-based study with a sample representative of the Korean population, we did not find any significant associations of gout with the two neurodegenerative diseases (AD and PD) after adjustment for not only age, sex, monthly income, and residential area but also body mass index (BMI), lifestyle factors, metabolic syndrome-related risk factors and comorbidities. The probabilities of AD and PD were only significantly higher in the gout group compared to the comparison group among individuals under the age of 60 years.

Recent epidemiological studies have reached inconsistent conclusions about the associations of gout with AD and PD. One study utilized claims data from a nationwide representative sample from Taiwan and revealed that patients with gout had a lower risk of both nonvascular (HR = 0.77, 95% CI = 0.72–0.83) and vascular dementia (HR = 0.76, 95% CI = 0.65–0.88)^9^. Similarly, according to a large population-based study, a 22% lower risk of AD was observed among individuals with higher serum uric acid levels (HR = 0.78, 95% CI = 0.66–0.91)^10^. A large longitudinal study, the Dutch Rotterdam study, also concluded that the risk of AD was lower among those with higher serum uric acid concentrations (HR = 0.89, 95% CI = 0.80–0.99)^11^. These previous studies reported an inverse association between gout and dementia, supporting the purported potential neuroprotective role of uric acid, a powerful antioxidant.

Conversely, other observational studies have shown that hyperuricemia is linked to a higher risk of dementia and cognitive decline^12,13^. A large French population-based study in people 65 years or older reported that hyperuricemia was associated with a higher risk of AD (HR = 1.55, 95% CI = 0.92–2.61) as well as age-related brain changes identified by MRI, demonstrating a clinical-pathological correlation^3^. In addition, one report using US Medicare claims data showed that gout was independently associated with a 15% higher risk of incident dementia in older adults (HR = 1.15, 95% CI = 1.12–1.18)^14^. These epidemiological data demonstrating a higher risk of dementia in patients with gout mainly focused on the role of uric acid in oxidative stress and inflammation.

As in our results, several other reports have also found no association between gout and AD. A recent study using 2-sample Mendelian randomization (MR) analysis, which is not susceptible to bias from unmeasured confounders or reverse causation, determined that epidemiological evidence did not support a causal relationship between gout and AD^15^. Other MR analyses also found no support of a causal role of genetically elevated serum levels of uric acid on the risk of AD (OR = 1.02, 95% CI = 0.93–1.12)^16^.

Likewise, epidemiological data regarding the role of high uric acid levels in the development of PD have provided inconsistent results. A number of studies have revealed strong evidence of an inverse relationship of serum levels of uric acid (or gout) with PD, which suggests that individuals with gout may have a lower risk of PD^17,18^. In other words, these observations highlight the potential involvement of oxidative stress in PD and the protective role of uric acid, an antioxidant, against the development of PD. On the other hand, a UK study found that in the gout group, the increase in the risk of subsequent PD was modest (risk ratio [RR] = 1.11, 95% CI 1.05–1.17)^19^. They suggested that the inflammatory state associated with the development of symptomatic gout or recurrent inflammatory arthritis may have counteracted any potential protective effect of uric acid as an antioxidant. Similar to our results, recent studies failed to find a robust association between gout and PD^20^. Two large MR analyses also reported no relationship between uric acid and the risk of PD, which suggests there is no clear causal association between serum levels of uric acid and the risk of PD^21,22^.

Because the present study had an observational design, we are unable to determine the plausible reason or mechanism underlying the lack of association between gout and neurodegenerative diseases. Based on previous research, possible biological explanations may involve the neutralization or incapacitation of two powerful functions of uric acid. Uric acid has antioxidative properties; specifically, uric acid scavenges reactive oxygen species^23^ and exerts a neuroprotective function by inhibiting oxyradical accumulation and preserving mitochondrial function, suppressing the cytotoxic action of lactoperoxidase, repairing DNA damaged by free radicals, and protecting against dopamine-induced apoptosis^24^. Gout flares occur due to the sudden release of monosodium urate crystal deposits in the joints, setting off an inflammatory cascade that manifests as an acute gouty arthritis attack^25^. Uric acid thus appears to be able to activate the immune response and, in that context, mediate the inflammatory process via the inflammasome^26^. Although this acute phase is self-limiting, one study found that monosodium urate crystals remain in the synovial fluid, causing persistent low-grade inflammation during the intercritical period^27^. As a result, acute and chronic inflammatory conditions of gout can yield both inflammatory responses and antioxidant effects, making it impossible to predict the associations of gout with AD or PD due to the interaction or counteraction between two competing actions.

Intriguingly, several studies (including a meta-analysis) reporting an inverse association between gout and incident PD described a stronger association in men than in women^28^. A recent study demonstrated that higher serum levels of uric acid were correlated with higher dopamine transporter uptake as assessed by a positron emission tomography (PET) scan, indicating a neuroprotective effect of gout against PD; this neuroprotective effect was more evident in women than in men^29^. Sex-specific analyses in a randomized placebo-controlled trial showed a correlation between an increase in serum uric acid levels and a slower rate of PD progression in women but not in men^30^. This sex difference in the association between gout and subsequent PD may suggest a greater neuroprotective effect of uric acid in women than in men. However, in sex-stratified analyses in the present study, this difference was not identified. Rather, in age-stratified analyses of both AD and PD probabilities, a positive correlation with gout was shown individuals under 60 years of age. Likewise, in the weight-stratified analysis of PD probability, a positive association with gout was found among participants with overweight. If gout is diagnosed at a relatively young age or when it is overweight, it may be assumed that the role of uric acid in the development of neurodegenerative diseases is more likely to be an inflammatory response than an antioxidant effect; but these postulations remain to be demonstrated, and further well-designed randomized controlled studies are needed to support this assumption.

This study should be interpreted with caution due to some limitations. First, this study was conducted using data extracted from a medical registry database, and diagnoses were defined based on diagnostic codes, which may increase concerns about inaccuracy of diagnoses of AD, PD, and gout due to codes. We further defined the presence of gout, AD, and PD as individuals with relevant claim codes based on at least two clinic visits, which may compensate for these shortcomings. Second, information on laboratory data was not available in this health insurance database; therefore, actual serum levels of uric acid were not included in the analyses. Third, we did not investigate drug use that could affect AD or PD, such as various gout management drugs, including uric acid-lowering drugs, nonsteroidal anti-inflammatory drugs (NSAIDs), steroids, interleukin (IL)-1 blockers, and colchicine. Further studies on the effects of gout medications are needed. Fourth, to identify the effect modification of age on the association between gout and AD or PD, interactions between age and gout should be tested using the Cox proportional hazard model with an added interaction term and covariates. Similarly, the same analysis is needed to test the effects of pooled normal and overweight on the risk of PD, but we could not consider them in this study. Finally, other unmeasured confounding factors, such as ethnicity, genetics or a familial history of neurodegenerative diseases, could not be completely excluded. Thus, it is possible that residual confounding effects and bias in the associations of gout with AD and PD may have been present.

In conclusion, our nationwide longitudinal follow-up study supports the possibility that gout does not significantly influence the occurrences of AD and PD. Additional evidence from future studies will complement these findings, further clarify these associations, and elucidate the pathophysiology of AD and PD.

## Methods

### Ethics

The Ethics Committee of Hallym University (2019-10-023) approved this study. The need for written informed consent was waived by the Institutional Review Board. All analyses in this study were performed in accordance with the guidelines and regulations of the Ethics Committee of Hallym University. The study utilizes data from the Korean National Health Insurance Service-Health Screening Cohort. A random sample of about 10% of the total population (approximately 515,000 out of 5,150,000) that underwent a health check-up between 2002 and 2003 was selected by the Korean National Health Insurance Service (NHIS). The age and sex specific distributions of the cohort population is available online. All Korean over the age of 40 and their families are required to undergo bi-annual health checks at no cost. The study benefits from the fact that all Korean citizens have a lifelong, 13-digit resident registration number, allowing for accurate population statistics to be calculated. This number is used in all hospitals and clinics in Korea and ensures that medical records do not overlap even if a patient moves. The Korean Health Insurance Review and Assessment (HIRA) system manages all medical treatments in Korea and the causes and dates of death, as recorded on death certificates by medical doctors, are officially reported. This NHIS data includes health insurance claim codes, diagnostic codes based on the International Classification of Disease-10 (ICD-10), death records, socioeconomic data, and health check-up information (including BMI, drinking and smoking habits, blood pressure, urinalysis results, hemoglobin levels, fasting glucose, lipid parameters, creatinine, and liver enzyme levels) for each participant from 2002 to 2013. Details regarding the Korean National Health Insurance Service-Health Screening Cohort data have been presented elsewhere^31^.

### Gout cases

According to the methods of a previous study^32^, gout cases were defined as participants who had visited the clinic or hospital more than twice with a diagnosis of gout according to the 10th revision of the International Classification of Diseases and Related Health Problems (ICD-10) code M10 (gout).

### Ascertainment of AD

Participants were considered to have AD if they were diagnosed with AD (ICD-10 code: G30) or Dementia in Alzheimer's disease (ICD-10 code: F00). To ensure the accuracy of diagnosis, we included only participants who had been treated ≥ 2 times for AD, as in our earlier studies^33^.

### Ascertainment of PD

Participants were considered to have PD if they were diagnosed with PD (ICD-10 code: G20). To ensure the accuracy of diagnosis, we recruited only participants who had visited hospitals or clinics ≥ 2 times for the treatment of PD, as in our previous study^34^.

### Selection of case and comparison groups

**Figure 3:**
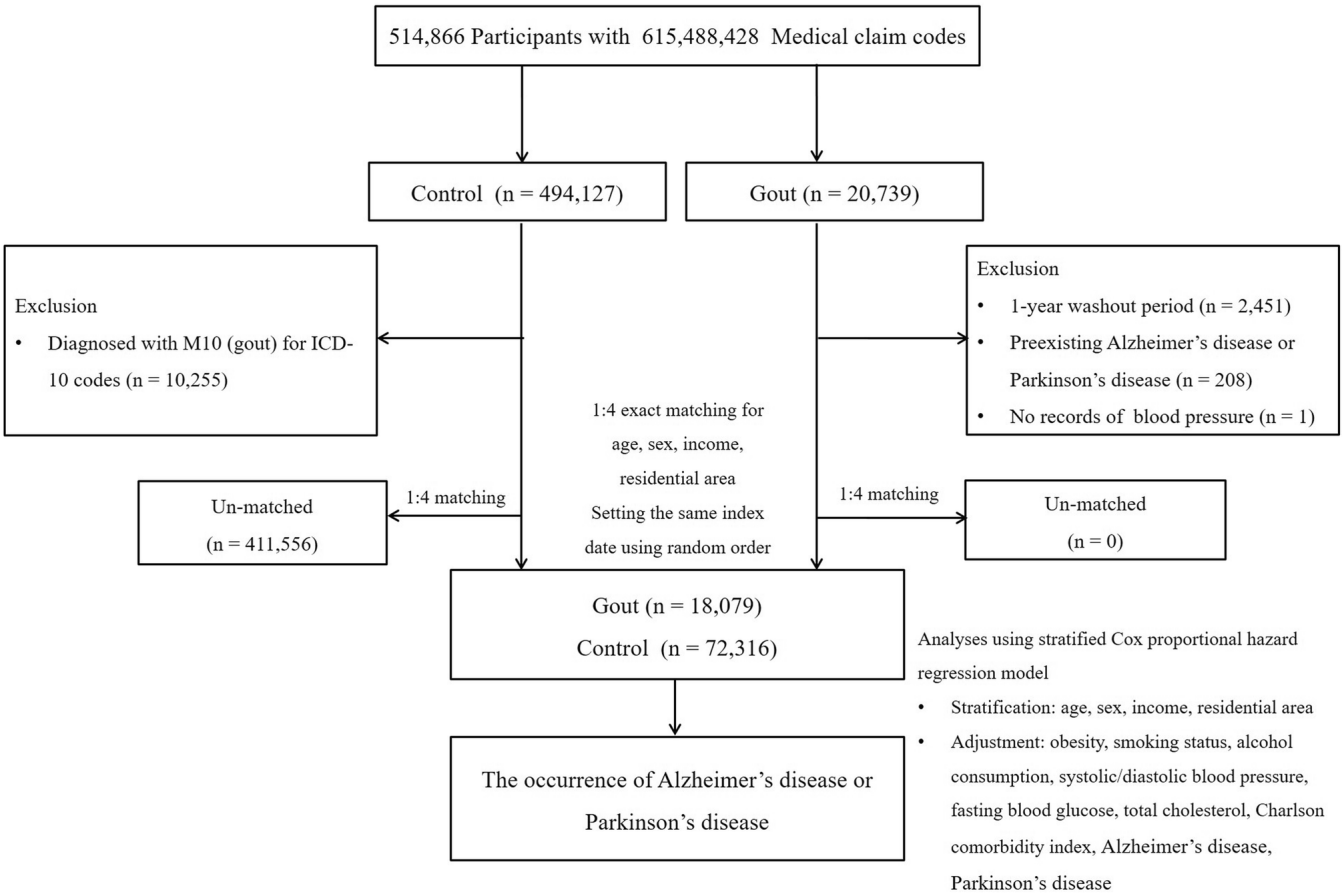
A schematic illustration of the group of participants selected for analysis of this study from the National Health Insurance database. Of the 514,866 participants, 18,079 gout participants were identified, and 72,316 control participants without gout were matched at a 1:4 ratio on age, sex, income, and residential area.

Gout participants were selected from 514,866 participants with 615,488,428 medical claim codes between 2002 and 2015 (n = 20,739); the remaining participants were included in the comparison group (n = 494,127). Individuals diagnosed with gout in 2002 (n = 2451) were excluded from the analysis such that the gout group consisted solely of participants first diagnosed with gout after 2002 (a washout period). Participants without blood pressure records in the gout group (n = 1) and those diagnosed with gout once previously in the comparison group (n = 10,255) were excluded. Gout participants were 1:4 matched with comparison participants in terms of age, sex, monthly income, and residential area (urban or rural). To limit selection bias in the matching process, participants without gout were randomly arranged and then included from the top of the list to the bottom. We assumed that comparison participants were evaluated simultaneously with each corresponding gout participant; in other words, that both participants had the same index date. Participants who died before the index date and those who had a history of AD or PD before the index date were excluded from each group. As a result of the matching process, 208 and 411,556 participants were excluded from the gout group and the comparison group, respectively, and thus, 18,079 gout participants and 72,316 comparison participants were included in the analysis (Fig. 3).

### Covariates

Participants were divided into 10 age groups at 5-year intervals and 5 income groups from class 1 (lowest income) to class 5 (highest income). The residential area was categorized as urban or rural following our previous study^35^. Smoking status, alcohol consumption, and obesity (using BMI, kg/m^2^) were categorized in the same manner as in our prior study^36^. The records of systolic blood pressure (SBP, mmHg), diastolic blood pressure (DBP, mmHg), fasting blood glucose (mg/dL), and total cholesterol (mg/dL) were also included in the analysis.

The Charlson Comorbidity Index (CCI), which assesses the burden of disease using 17 comorbidities, was calculated as a continuous variable from 0 (no comorbidities) to 29 (multiple comorbidities; excluding dementia) in the analysis.

### Statistical analyses

The standardized difference in means was used to evaluate whether the distribution of baseline characteristics between the gout and comparison groups was balanced after matching. Stratified Cox proportional hazard models were used to examine the associations of gout with AD or PD. Crude (unadjusted) models included participants matched by age, sex, income, and residential region. The adjusted models controlled for obesity, smoking status, alcohol consumption, SBP, DBP, fasting blood glucose, total cholesterol, CCI, AD diagnosis and PD diagnosis and provided HRs and 95% CIs. We also used the Kaplan–Meier estimates to map the survival curves of the gout and comparison groups, and differences in their survival rates were evaluated with a log-rank test.

In addition, we further carried out subgroup analyses to examine the effects of various covariates on probabilities of AD and PD in patients with gout. Stratified Cox proportional hazard models were performed for age (< 60 years old and ≥ 60 years old) and sex. Unstratified Cox proportional hazard models were used for obesity (underweight, normal weight, overweight and obese), blood pressure (normal and hypertension), fasting blood glucose (< 100 mg/dL and ≥ 100 mg/dL), and total cholesterol (< 200 mg/dL and ≥ 200 mg/dL).

Two-tailed analyses were conducted, and the significance threshold was set at P values less than 0.05. SAS version 9.4 (SAS Institute Inc., Cary, NC, USA) was used for statistical analyses.

### Ethic declarations

The Ethics Committee of Hallym University (2019-10-023) approved this study.

## Data Availability

Releasing of the data by the research is not allowed legally. All data are available from the database of National Health Insurance Sharing Service (NHISS) https://nhiss.nhis.or.kr/ NHISS allows access to all of this data for the any researcher who promises to follow the research ethics at some cost. If you want to access the data of this study, you can download it from the website after promising to follow the research ethics.
